# Monthly Summary

**Published:** 1880-11

**Authors:** 


### MONTHLY SUMMARY.

*Unerupted Teeth in the Roof of the Mouth—Freaks of Nature.*—Sometimes we come across strange freaks of nature, things which border closely on mysteries, and to which it is exceedingly difficult to assign reasons. These things frequently come under the observation of the physician, and quite as frequently do they appear to the observing dentist. As to the truth of the old saying that "Facts are stranger than fiction," I give the following cases as examples: A few months ago a gentleman called to consult me in regard to a swelling in the roof of his mouth, near the median line, which was very sore and painful. The history of the case, as he gave it, was that about one year previous his artificial set of teeth commenced to rock, and to remedy it he cut out a small portion of the palatal side, which gave temporary relief; when it again would cause him discomfort, then he would repeat it, until he nearly cut through the plate. During all of this time it became more painful, so he could hardly sleep; this was accompanied by a slight discharge. On consulting his family physician, he pronounced it necrosis of the palatine surface of the maxilla bone, but referred him to me. On inserting a small silver probe I felt convinced that it was an unerupted tooth. Acting on this theory, I made a longitudinal incision through the soft parts, and with a straight pair of root forceps got a firm hold and brought it out, requiring considerable force to dislodge it. One of the peculiarities was that it came root downward, and having met with some obstruction was turned nearly at right angles posteriorly, the crown pointing towards the nares; there was some necrosis at the margins of bone, which was easily removed, after which it healed readily, presenting now a normal condition. Considerable hemorrhage accompanied the operation; patient of a bilious-lymphatic temperament; age forty-three years.

I am convinced that this is a case of third dentition, and for this reason: The gentleman has worn artificial dentures about eleven years; for the first seven, a partial plate with molars, bicuspids and one canine. I removed his remaining natural teeth, about four years ago, in order to insert a full denture, and am sure that the centrals were of the second dentition. I extracted them for absorption of the alveolus caused by the partial plate. The tooth being turned at nearly right angles, I think that it must have come in contact with some object before it was thoroughly ossified.

I will give a brief history of another case which came under my notice. A year or so ago a lady consulted me in reference to her plate, which, she said, caused her a great deal of annoyance from becoming loose and not fitting. She said that she had had a number of plates made, and for a time they would fit nicely, and then would commence to rock and trouble her. On making an examination, I thought I discovered the cause in a root, which I attempted to extract, when I found that there was something more. On cutting away the soft tissue, I found the point of an unerupted tooth; but so firmly was it embedded in the bone that I could get no hold of it whatever, so had to cut away a portion of the bone, which was easily done by the burring engine. I was then enabled to get a firm hold, and brought out a fully developed canine tooth one and one-fourth inches in length. It was laying in a nearly vertical position, the crown pointing toward the left, and the root toward the right. These cases are of interest in showing how wonderfully productive nature is in supplying germs of teeth.—*Dr. J. Allen Osmun, in Miscellany.*

---

*Another Discovery.*—Prof. Swift, Astronomer of the Warner Observatory, at Rochester, N. Y., discovered another large comet on the evening of October 10th. The fact was noted in the associated press dispatches, but some important and interesting details which could not be telegraphed are herewith given. The new celestial visitor is in the Constellation of Pegasus, right assention, 21 hours, 30 minutes, declination North 17 degrees, 30 minutes. Its rate of motion is quite slow, being in a North-westerly direction, so that it is approaching the sun. It has a very strong condensation on one side of the centre, in addition to a starlike neucleus, which indicates that it is throwing off an extended tail. From the fact of its extraordinary size, we are warranted in presuming that it will be very brilliant, and the additional fact that it is coming almost directly toward the earth, gives good promise that it will be one of the most remarkable comets of the present century. This is the fifth comet which Prof. Swift has discovered, and the increased facilities which Mr. H. H. Warner, the popular and wealthy medicine man, has given him, by erecting a magnificent Observatory for his benefit, promise much more for the future. There is a possibility that further developments may prove this to be the great comet of 1812, which is being constantly expected, in which event astronomers will have an unusual opportunity to test the spectroscope for the first time upon these eccentric bodies, and ascertain certainly what they are.

*Behavior of Metals When Heated in Vacuo.*—Thos. A. Edison read a paper before the American Association for the Advancement of Science on the above subject, the very interesting results of which we give in a condensed form:

A platinum wire of the five-thousandths of an inch in diameter, and weighing 343 milligrammes, lost, on being kept for nine hours at a moderate incandescence, 42 milligrammes.

By repeating the experiment under a glass shade, the sides of the shade were found coated with a film of platinum.

By varying the experiments he arrived at the conclusion that this effect was due, not to volatilization as commonly understood, but to a wearing away of the surface of the platinum by the impact of the stream of gases upon the highly incandescent surface. This was confirmed by repeating the experiment in vacuo, when the wire could be kept for hours at a dazzling glow without occasioning the slightest deposit on the glass.

By placing a platinum wire, thus treated, under the microscope, its surface will be found covered with innumerable cracks. This cracking is due entirely to the expansion of the air enclosed in the pores of the platinum, and subsequent contraction upon the escape of the air.

By subjecting a wire in vacuo to the electric current, at first weak and slowly increasing, the air will pass out gradually and without explosion. If the wire now be allowed to cool at intervals of fifteen minutes, increasing the current each time, it will be welded together by this expansion and contraction and become homogeneous, and viewed under the microscope will show no cracks, but present a highly polished, silvery white surface. It will be found to be exceedingly difficult to melt in the oxyhydrogen flame, not to be annealed at any known temperature, and give a light eight times stronger than a similar wire of platinum in the ordinary state.

Common iron wire treated in the same way may be made to give a light greater than platinum not treated.

Incidentally Mr. Edison found that the melting point of many oxides is dependent upon the manner of applying the heat.

He further thinks that the volatility of platinum above mentioned might be made use of to coat large plates of glass economically by placing them on each side of a large sheet of the metal kept incandescent by the electric current.—*Druggists Circular.*

---

*Disturbances of Vision Produced by Poisoning from Unsound Meat.*—We learn from the Edinburgh *Medical Journal*, for April, 1880, that Prof. H. Cohn, of Breslau, has observed two instances of poisoning, distinctly referable to the eating of game pie and pike which had been kept too long. The symptoms in both cases were severe gastric irritation, with vomiting and faintness, inability to swallow, and paresis of accommodation. In the first case a landlady had attempted to preserve the remains of a hare pie, of which a number of her customers had partaken with impunity. On tasting a small portion of this, some time afterward, she was seized within an hour with the most violent vomiting and other symptoms of poisoning. The next morning she was unable to read, although she had been able to do so the day before. She was first seen by Cohn nine days afterward, when the accommodation was found to be almost completely abolished. She recovered after a month. In the second case three members of a household, numbering six in all, were poisoned, three escaping, although it was ascertained that the symptoms came on after eating pike, and could be due to nothing else. All had, however, partaken of the same dish. On careful questioning it was found that the dish contained two fish, each of which was cut into three pieces, and it was conclusively shown that only those who had eaten the one (which was not perfectly fresh) were affected. In one the paresis of accommodation was the only symptom; the other two had severe gastric irritation besides. In one only the accommodation had not returned when last seen, six weeks after the occurrence. Although a number of instances of fish poisoning are on record, it does not appear that this curious local paralysis has been noted, though from remarks made in some instances, it appears probable that something similar existed. The other symptoms have always been the same. Cohn is inclined to ascribe it to some particular form of decomposition. —*Med. & Surg. Reporter.*

---

*Spence's Metal.*—A new metalic compound, which is adaptable to a great variety of uses, and is likely to prove one of the most valuable substances we have, has been lately brought into notice. It is called Spence's metal, and is formed by combining a metalic sulphide—iron pyrites, for instance, or other similar compound—with melted sulphur. The combination forms a liquid, which, on cooling, becomes a solid homogeneous mass, requiring a temperature of only 320° to melt it, and having the properties of expanding on cooling, of resisting atmospheric and climatic influences, acids, alkalies and water, and of being susceptible of a high polish. It is easily prepared for the mold by reason of its low melting point, and gives a nearly perfect cast. The range of purposes for which it may be used, is nearly as varied as that of India-rubber; and it is cheap, costing only one-fourth as much as lead, and demanding only the cheapest processes.—*Items of Interest.*

*The Value of Prolonged Artificial Respiration.*—The *Medical Press and Circular*, March 31, 1880, informs us that in a recent communication to the French Academy, Professor Fort raises again the question of premature interments. One fact he mentions is, that he was enabled to restore to life a child three years old, by practicing artificial respiration on it four hours, commencing three hours and a half after apparent death. Another case was communicated to him by Dr. Fournol, of Billancourt, who, in July, 1878, reanimated a nearly drowned person after four hours of artificial respiration. This person had been in the water ten minutes, and the doctor arrived one hour after asphyxia. Professor Fort insists also on the utility of artificial respiration in cases of poisoning, in order to eliminate the poisons from the lungs and glands. The length of time it is desirable to practice artificial respiration in any case of apparent death from asphyxia, Professor Fort has not yet determined, but his general conclusion is that it should be maintained perseveringly for several hours.—*Med. & Surgical Reporter.*

---

*Glass Wicks for Lamps.*—According to a German trade paper, a new kerosene and spirit lamp has been invented, in which the wick is made entirely of glass. Several advantages are claimed for it. The flame clings closely to the wick, so that lighted lamps may be carried about without fear of their being extinguished by sudden draughts; moreover, no sparks are liberated from it. With an equal amount of this wick turned up, a much brighter and clearer light is obtained than where cotton ones are used. The smoking is greatly reduced, and at least 10 per cent. of oil is saved. There is scarcely any waste of the wick itself, and the troublesome trimming and cutting to which lamp burners are accustomed are altogether needless, for no portion of it is carbonized. Used in spirit lamps it greatly increases the heat of the flame, and finally, it is claimed that it can be produced and sold at a cheap rate.

---

*Anæsthetics in* 1651.—The *Lancet* informs us that in a recent work, entitled "Histoire de la Medicine a Troyes," Dr. Guichet relates that the College of Physicians of that town brought an action against a certain Nicolas Bailli for administering internal remedies to his patients and *putting them to sleep.* In defence Bailli declared that having observed that in great operations, amputations, incisions, actual and potential cauterizations, many patients slipped through his hands for want of sleep, he had studied the secrets of nature, and had at last found a cordial, or marvelous essence, which put them to sleep softly, and appeased their sensibility to pain.—*Med. & Surg. Reporter.*

*Motion in Teeth.*—Teeth appear to be motionless—immovably fixed in the jaws—but they are not. They are in constant motion and swaying to and fro, backward and forward, and grinding and chafing against each other without intermission. Of course this is very slight, perfectly imperceptible to the eye and touch, except their abnormal action—motion in excess—which is evinced by disease, as in inflammation of a nerve, ulceration, salivation, &c.

There are several proofs of our position. Among them are two which must have come under the observation of most dentists.

Perhaps you never attempted to fill two teeth solidly together. I would'nt acknowledge it if I had; it is too unprofessional. But I presume you have seen such a case. But did you ever see such an one which was successful for a year? The filling from one or the other broke away, though it may have had to bring a piece of the tooth with it; or else in their struggles both become sensibly loose. Did you ever cap two or more teeth together to prevent the occlusion of others in the regulation of teeth? Then you have in this way discovered this same peculiar irresistible power that almost defied your effort to confine the bound teeth to stationary positions.—*Items of Interest.*

---

*Nitro-Glycerin.*—Nitro-glycerin can be prepared for use in a solution either of alcohol or ether, a one per cent. solution in alcohol being that generally preferred. It being also soluble in melted cocoa-butter, this has been employed for making it into pills, but in this form it does not act so quickly as in alcoholic solution. A better plan than this is to add an equal quantity of chocolate paste to this pill mass, and then make it up into lozenges of any desired size, which it would not be disagreeable to chew up in the mouth, and thus gain rapid absorption. Nitro-glycerine bids fair to prove an anti-neuralgic of the greatest value, especially in cases of angina pectoris and such other affections as nitrate of amyl has hitherto been much used in, taken at intervals of two to four hours, in doses of about two drops of the one per cent. solution, and increasing until full physiological action is obtained. The relaxed condition of the blood vessels induced lasts usually about half an hour. Doses of the above strength and frequency have been continued through several consecutive days with safety and success in warding off threatening attacks of angina pectoris in cases in London hospitals.—*Louisville Medical News.*

